# Supplementary material for: The genome sequence of the Scalloped Oak, Crocallis elinguaria (Linneas, 1758)
Source: Wellcome Open Res. 2023 Oct 6;8:426. [Version 1] doi: 10.12688/wellcomeopenres.20081.1 (PMC11031643; doi:10.12688/wellcomeopenres.20081.1)
Supplement: Peer review history

# Reviewer report: Reviewer response for version 1

**DOI:** 10.21956/wellcomeopenres.22239.r78876

**Published:** 2024-04-19

**Authors:** Yuttapong Thawornwattana

**Affiliations:** 1 Harvard University, Cambridge, Massachusetts, USA

Boyes & Boyes present a chromosomally complete assembly of *Crocallis elinguaria *(Linneas, 1758) (Lepidoptera: Geometridae), a moth collected from Wytham Woods, England.  It was generated primarily PacBio HiFi, 10X and Hi-C data.

 The assembly is of high quality and will be useful to the research community. It contains 15 autosomes, the Z and W chromosomes and the mitochondrial genome. It is impressive to have the W chromosome in the assembly, but it is unclear in the Hi-C contact map (fig 5) which scaffold might corresponds to the W.  Having chromosome labels in the figure would be useful.  It would be nice to mention the expected haploid chromosome number, for example from cytology studies, to confirm that the chromosomes recovered here represent the complete genome.

Are sufficient details of methods and materials provided to allow replication by others?

Yes

Is the rationale for creating the dataset(s) clearly described?

Yes

Are the datasets clearly presented in a useable and accessible format?

Yes

Are the protocols appropriate and is the work technically sound?

Yes

Reviewer Expertise:

evolutionary genomics, phylogenetics, insect evolution

I confirm that I have read this submission and believe that I have an appropriate level of expertise to confirm that it is of an acceptable scientific standard.

# Reviewer report: Reviewer response for version 1

**DOI:** 10.21956/wellcomeopenres.22239.r76939

**Published:** 2024-04-03

**Authors:** Laurence Despres

**Affiliations:** 1 Univ. Grenoble-Alpes, Grenoble, France

In this data note, the authors report on the  chromosome-level genome assembly of the Scalloped Oak, *Crocallis elinguaria *(Lepidoptera; Geometridae). a common and widely spread yet declining Eurasian wood moth. The assembly appears to be of good quality using appropriate methods : Pac Bio HiFi long reads (44-fold coverage), 10X genomics (103 fold-coverage) and Hi-C data to confirm scaffolding, manual curation of the final assembly, and following the high standard of the Darwin Tree of Life Project for results presentation.

 The final assembly has a total length of 430.4 Mb and includes 17 chromosomes: 15 autosomes and the sexual Z and W chromosomes, as expected given that the specimen sequenced is a female. The mitogenome was also assembled. The quality of the assembly was further assessed by the proportion of complete BUSCO genes recovered, which is very high (>98% of Lepidoptera database BUSCO genes). Annotation was performed using BREAKER3 and 17,741 protein-coding genes were predicted.

 I have only one minor suggestion: Given the large range of colour variation described for this species you might highlight in the introduction that the specimen sequenced corresponds to the most common form by referring to the photo of the specimen presented in figure 1 at the end of this sentence: ‘... but the most common form is yellowish-buff with a darker brown central bar ornamented by a single black spot’ (see figure 1).

Are sufficient details of methods and materials provided to allow replication by others?

Yes

Is the rationale for creating the dataset(s) clearly described?

Yes

Are the datasets clearly presented in a useable and accessible format?

Yes

Are the protocols appropriate and is the work technically sound?

Yes

Reviewer Expertise:

population genomics

I confirm that I have read this submission and believe that I have an appropriate level of expertise to confirm that it is of an acceptable scientific standard.
